# 吉西他滨联合长春瑞滨方案一线治疗中国晚期非小细胞肺癌患者的多中心回顾性研究

**DOI:** 10.3779/j.issn.1009-3419.2012.05.06

**Published:** 2012-05-20

**Authors:** 永峰 虞, 力 张, 志生 任, 九军 赵, 钟瑞 李, 舜 陆

**Affiliations:** 1 200030 上海，上海交通大学附属胸科医院肺部肿瘤临床医学中心 Shanghai Lung Cancer Center, Chest Hospital Affiliated to Shanghai Jiaotong University, Shanghai 200030, China; 2 510060 广州，中山大学肿瘤医院肿瘤科 Department of Medical Oncology, Cancer Center of Sun Yat-Sen University, Guangzhou 510060, China; 3 130000 长春，吉林省人民医院胸外科 Department of Thoracic Surgery, Jilin Province People's Hospital, Changchun 130000, China; 4 163001 大庆，大庆市油田总医院肿瘤科 Department of Medical Oncology, Daqing Oilfield General Hospital, Daqing 163001, China; 5 710068 西安，陕西省肿瘤医院内科 Department of Medical Oncology, Shaanxi Cancer Hospital, Xi'an 710068, China

**Keywords:** 吉西他滨, 长春瑞滨, 一线治疗, 肺肿瘤, Gemcitabine, Vinorelbine, First-line treatment, Lung neoplasms

## Abstract

**背景与目的:**

化疗是目前晚期非小细胞肺癌主要的治疗手段，非铂两药方案因避免了严重的肝肾毒性，成为常见的治疗选择。本研究回顾性分析晚期非小细胞肺癌一线使用吉西他滨+长春瑞滨（GN）方案的患者，探讨该方案的疗效和安全性。

**方法:**

通过非干预的方式收集国内5家医院在2004年1月1日-2010年6月30日行GN方案一线治疗的晚期非小细胞肺癌患者，统计患者的疗效及毒副反应。应用*Kaplan-Meier*法进行生存分析，*Cox*方法对影响患者疗效的年龄、性别、分期、病理类型等进行多因素分析。

**结果:**

共67例患者采用了GN方案，52例为RRM1阴性和ERCC1阳性患者，其余15例患者未行RRM1和ERCC1测定。其一线治疗的客观缓解率为34.3%，疾病控制率为76.1%，中位无进展生存期为5.5个月，中位总生存期为22.1个月，多数患者的毒副反应可以耐受。体能状况评分、是否后续治疗对患者的生存期有明显影响。

**结论:**

GN方案在晚期非小细胞肺癌一线治疗中有较好的疗效和安全性。

## Abstract (en)

**Background and objective:**

Chemotherapy has become the mainstay of first-line therapy. Non-platinum containing drugs are characterized by favorable toxicity profiles and is better tolerated than platinum-based regimens. The aim of this study is to detect the efficacy and toxicity of gemcitabine and vinorelbine (GN) in advanced non-small cell lung cancer (NSCLC) first-line treatment in China.

**Methods:**

We retrospectively reviewed 67 NSCLC patients treated with this agent at five Hospital in China from Jan 2004 to Jun 2010. Survival analysis was evaluated by *Kaplan-Meier* method, multivariate analyses were performed to find prognostic markers using *Cox* proportional hazards model.

**Results:**

A total of 67 patients were analyzed in this study. There were 52 patients with RRM1 negative and ERCC1 positive. The objective response rate was 34.3%. The disease control rate was 76.1%. The progression-free survival and median overall survival was 5.5 and 22.1 months, respectively. On multivariate analysis, performance status score and whether further treatment were independent prognostic factor for overall survival.

**Conclusion:**

The GN agent is effective for the first line treatment in advanced non-small cell lung cacer. The toxicity is well tolerated.

非小细胞肺癌（non-small cell lung cancer, NSCLC）是导致癌症死亡的首要原因^[1]^。由于40%-50%的NSCLC患者确诊时已属晚期，化疗是目前针对晚期NSCLC的延长生存期的主要手段。

目前标准的一线化疗方案为含铂两药联合化疗^[2-4]^。ECOG1594^[5]^显示三代药物联合铂类相比在有效率和生存率方面均未见差异。尽管含铂方案目前成为一线治疗的标准方案，由于铂类药物在肾脏、胃肠道、耳、血液学等方面的毒性反应，成为临床医生在用药过程中特别需要关注的问题。因此研究人员试图通过其它有效药物的组合来达到同含铂方案治疗相同或相近的效果。非铂方案试图降低含铂药物所带来的胃肠道反应、肝肾毒性等不良反应，且非铂方案的组合多选择两种毒性反应不同的药物。吉西他滨+长春瑞滨（GN方案）是目前临床试验中常用的非铂方案。大型Ⅲ期临床试验^[6, 7]^显示，GN方案在一线晚期NSCLC方面其有效率约为25%-41%，与含铂两药方案相比在有效率和生存期方面无明显差异，但毒性反应明显降低。与单药方案相比则提高了有效率和生存时间。因此，GN方案是一种比较理想的化疗组合，特别是近年来关于ERCC1等分子标志物研究进展表明，ERCC1表达阳性的患者不能从含铂方案中获益，因此，基于ERCC1等分子标志物指导下的非铂方案特别是GN方案的组合便成为较好的选择。

本研究回顾性收集国内多家医院既往采用GN方案治疗晚期NSCLC治疗的相关数据，试图探讨国内GN方案一线治疗晚期NSCLC的疗效及安全性，为临床诊疗提供参考。

## 1 资料与方法

### 1.1 临床资料

收集2004年1月1日-2010年6月30日在国内5家医院就诊的具有完整随访资料的Ⅲb期和Ⅳ期晚期NSCLC患者。一线治疗中共67例患者使用了GN方案，患者一般特征见表 1。

**1:** 患者的一般特征及PFS单因素分析 Demographic characteristics and the univariate analysis of PFS of the patients

	*n* (%)	PFS (month)	95%CI	*P*
Gender				0.36
Male	43 (64)	5.6	3.2-7.9	
Female	24 (36)	4.8	2.6-6.9	
Age (year)				
Range	32-78			
Median	60			
< 65	48 (72)	4.7	3.6-5.8	0.557
≥65	19 (28)	6.8	4.4-9.2	
Stage				0.573
Ⅲb	6 (9)	5.1	0-10.6	
Ⅳ	61 (91)	5.5	4.0-7.0	
PS score				0.035
0-1	52 (78)	5.6	4.9-6.2	
2	15 (22)	5.1	2.0-8.2	
Histology				0.057
Adenocarcinoma	46 (69)	4.8	3.9-5.7	
Squamous cell carcinoma	21 (31)	8.1	5.9-10.3	
Smoking history				0.55
Yes	42 (63)	5.6	3.2-7.9	
No	25 (37)	5.1	4.4-5.7	
ERCC1/RMM1 detected				0.058
Yes	38 (57)	5.6	4.3-8.2	
No	29 (43)	4.7	2.9-5.9	
PFS: progression free survival; PS: performance status.				

### 1.2 治疗方法

使用吉西他滨（商品名：泽菲）+长春瑞滨（商品名：盖诺）（江苏豪森药业生产）联合方案化疗。吉西他滨1, 000 mg/m^2^-1, 250 mg/m^2^静脉滴注30 min，长春瑞滨25 mg/m^2^静脉滴注15 min-20 min。每21 d-28 d为1个周期。两组均不使用预防性升白细胞治疗。治疗期间根据需要使用粒细胞集落刺激因子、白介素11、止吐、抗感染等对症支持治疗。

### 1.3 疗效及毒性反应评定标准

根据实体瘤疗效评价标准（Response Evaluation Criteria in Solid Tumors, RECIST）评价近期疗效，分为完全缓解（complete response, CR）、部分缓解（partial response, PR）、疾病稳定（stable disease, SD）和疾病进展（progressive disease, PD）。客观有效率（objective response rate, ORR）＝（CR+PR）/（CR+PR+SD+PD）×100%。疾病控制率（disease control rate, DCR）＝（CR+PR+SD）/（CR+PR+SD+PD）×100%。定期复查血常规、肝肾功能、心电图、B超及CT，每2个化疗周期或临床考虑疾病进展时评价疗效。

### 1.4 RRM1与ERCC1检测

67例患者中，52例患者进行了RRM1与ERCC1检测，用山羊抗人多克隆RRM1抗体及小鼠抗人单克隆ERCC1抗体进行免疫组化实验。各医院检测方法及结果判读由本院病理科医师完成。检测结果均为ERCC1阳性、RRM1阴性。

### 1.5 随访和生存分析

随访由各家医院采用门诊或电话方式，末次随访时间为2011年9月1日。总生存期（overall survival, OS）定义为患者自首次治疗起至患者死亡或末次随访的时间。无进展生存期（progression free survival, PFS）定义为患者自GN方案治疗开始至明确为PD的时间。

### 1.6 统计学分析

应用SPSS 17.0软件进行统计学分析。计数资料的比较采用χ^2^检验，应用*Kaplan-Meier*法进行生存分析，对影响患者疗效的年龄、性别、分期、病理类型等因素进行*Cox*多因素分析。*P* < 0.05为差异有统计学意义。

## 2 结果

### 2.1 近期疗效

67例患者均可评价疗效，所有患者均完成至少1个周期的治疗，中位治疗周期数为3（范围1-6），所有患者均可评价疗效，其中CR 1例，PR 22例，SD 28例，PD 16例，ORR为34.3%，DCR为76.1%。

### 2.2 PFS和OS

中位随访时间为26.8个月。67例患者获得了完整的PFS时间，64例患者获得了OS时间，其中3例患者失访。所有患者的中位PFS时间为5.5个月（图 1），64例完整随访的患者的中位OS时间为22.1个月（图 2）。

**1:**
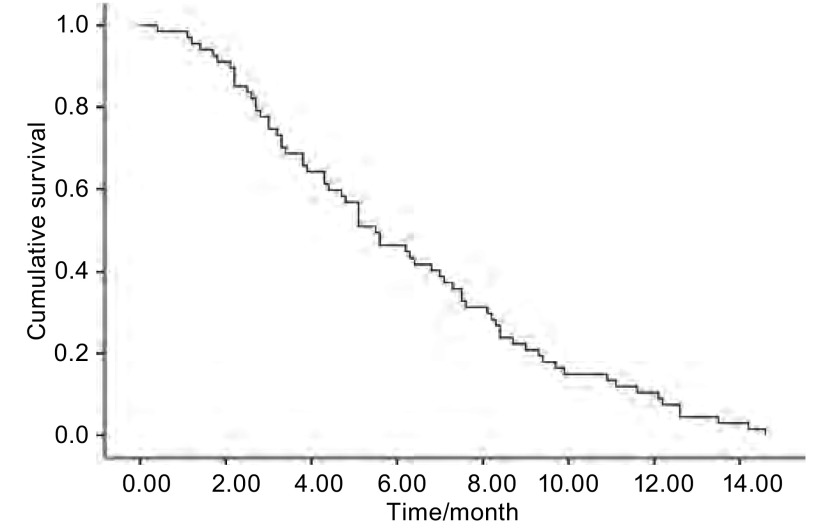
67例晚期非小细胞肺癌患者一线GN方案的PFS The PFS curves of 67 patients received GN regimen therapy

**2:**
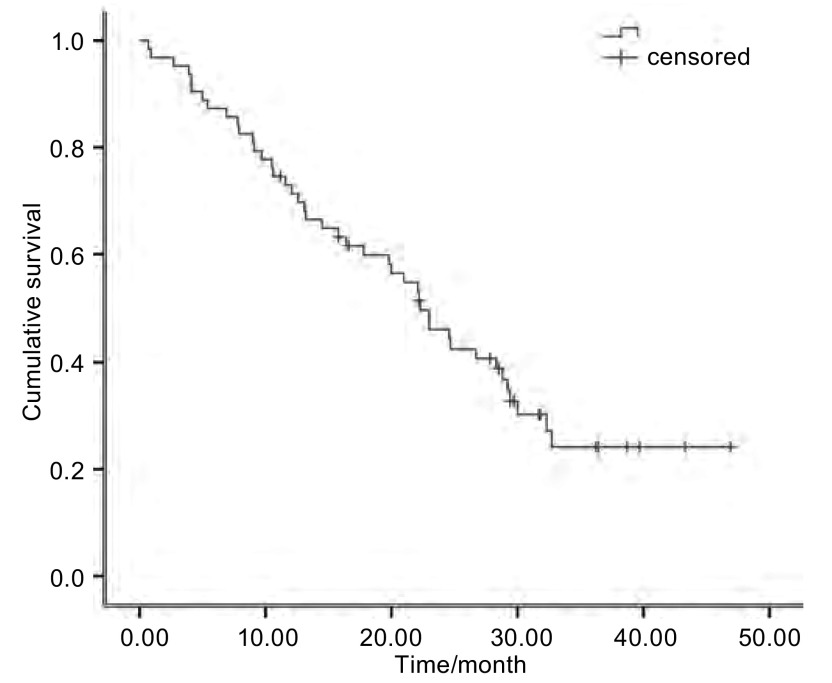
64例完整随访的患者的总生存期 The overall survival curves of 64 patients received GN regimen therapy

### 2.3 影响患者PFS的单因素分析及总生存期的多因素分析

无论患者性别、年龄、分期、病理类型、吸烟状况等均显示PFS无统计学差异，但体能状况（performance state, PS）评分为0分-1分的患者PFS明显好于PS评分为2分的患者（*P*=0.035）（图 3）。对于明确ERCC1(+)/RRM1(-)的患者的PFS时间长于未进行相关检测的患者，具有统计学差异（表 1）。共43例患者在一线治疗进展后进行了二线治疗，43例患者中化疗患者22例（含铂两药方案8例，单药方案14例），靶向治疗21例（厄洛替尼9例，吉非替尼12例）。对影响患者生存期的因素分析显示，PS评分与是否进行后续治疗可影响患者预后，PS评分差和未进行后续治疗是影响患者生存期的不良因素（表 2）。

**3:**
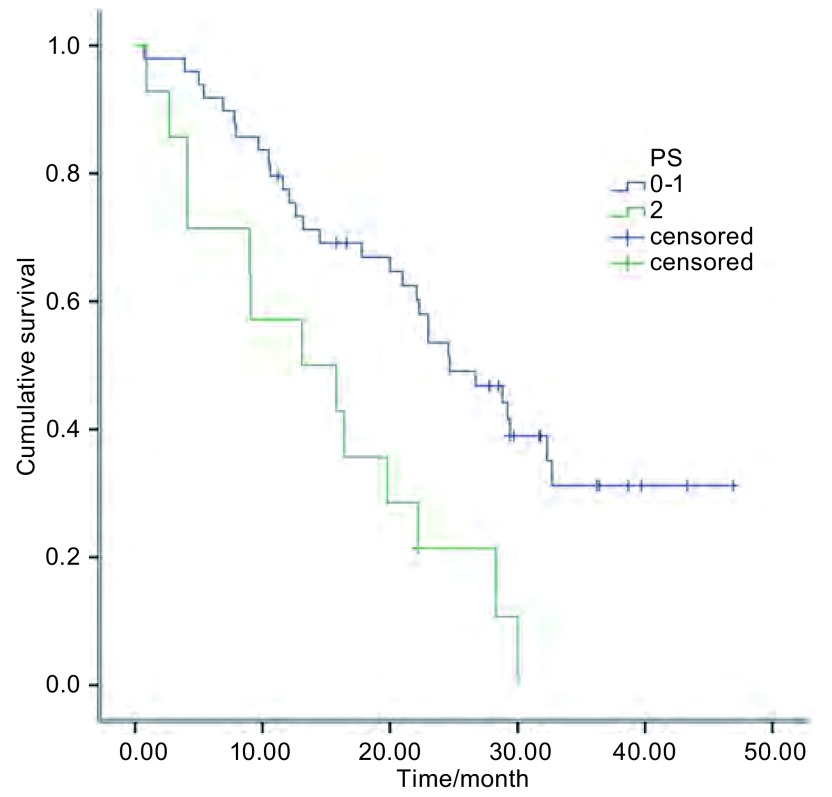
PS评分为0分-1分的患者和2分患者的总生存期比较 *Kaplan-Meier* curves of overall survival for patients in PS with 0 to 1 and 2

**2:** 影响患者生存期的多因素分析 The relationship between overall survival and clinical chracteristic of 67 patients analyzed by *Cox* multivariate model

Variable	HR	95%CI	*P*
Gender	0.71	0.21-2.40	0.580
Age	0.62	0.29-1.230	0.220
Staging	2.90	0.69-6.28	0.140
PS score	2.70	1.30-5.90	0.012
Histology	2.20	1.30-3.90	0.097
Treatment after progression	0.61	0.32-0.92	0.038
Smoking history	1.70	0.50-5.10	0.365
ERCC1/RMM1 detected	2.10	0.95-3.58	0.135

### 2.4 不良反应

多数患者的毒副反应为Ⅰ度-Ⅱ度，发生Ⅲ度-Ⅳ度不良反应的患者比例较低，其中，厌食、静脉炎和恶心呕吐发生比例相对较高。未发生因毒副反应停药的患者。血液学毒性方面，中性粒细胞降低较为常见，其中Ⅰ度-Ⅱ度发生比例为29.8%，15例患者发生了Ⅰ度-Ⅱ度血小板降低。其它不良反应均在可控制范围内（表 3）。

**3:** 患者主要不良反应 The main toxicity of all the patients

Toxicity	Grade Ⅰ-Ⅱ toxicity	Grade Ⅲ-Ⅳ toxicity
Nausea /Vomiting	22 (32.8%)	2 (2.9%)
Anorexia	24 (35.8%)	4 (5.9%)
Alopecia	20 (29.9%)	9 (13.4%)
Dyspnoea	9 (13.4%)	5 (7.5%)
Diarrhoea	6 (8.96%)	2 (2.9%)
Neurotoxicity	3 (4.48%)	1 (1.5%)
Cough	8 (11.9%)	4 (5.9%)
Constipation	14 (20.9%)	5 (7.5%)
Pyrexia	8（11.9%）	1 (1.5%)
Stomatitis	17 (25.4%)	5 (7.5%)
Myalgia	9 (13.4%)	2 (2.9%)
Thrombocytopenia	15 (22.4%)	4 (5.9%)
Neutropenia	20 (29.9%)	9 (13.4%)
Anaemia	12 (17.9%)	3 (4.5%)
ALT/AST	15 (22.4%)	3 (4.5%)
Phlebitis	20 (29.9%)	3 (4.5%)

## 3 讨论

本研究通过回顾性分析国内多家医院的GN方案一线治疗疗效显示，其ORR达34.3%，DCR达76.1%，中位PFS为5.5个月，且毒副反应相对较轻。

吉西他滨是盐酸吉西他滨为核苷同系物，属细胞周期特异性抗肿瘤药^[8]^。主要杀伤处于S期（DNA合成）的细胞，同时也阻断细胞增殖由G_1_期向S期过渡的进程，单药疗效19%-27%，长春瑞滨主要作用于G_2_期和有丝分裂（M）期，抑制微管聚合，使分裂细胞不能形成纺锤体，单药有效率14%-33%^[9]^。两药联合在理论上能够起到协同作用。Gridelli^[6]^进行的一项随机临床试验共入组501例患者，一组为GN方案，另外一组为NP或GP方案，结果显示，两组中位进展时间及中位OS均未见明显差异。Kuzur等^[10]^研究显示，GN方案较单药吉西他滨、紫杉醇疗效有所提高。本研究的结果无论在疗效还是在毒副反应方面均与既往研究相近。

个体化化疗是当前临床研究的热点，针对不同的耐药基因的检测可以更好地针对性使用化疗药物，提高化疗疗效的同时避免毒副反应的产生。*ERCC1*和*RRM1*是DNA修复基因。ERCC1定位于19号染色体上，是核苷酸剪切修复家族中的一个重要成员，编码297个氨基酸的蛋白，与XPF形成异源二聚体，在DNA单链受损处发挥功能^[11]^。RRM1是核苷酸还原酶调节M1亚单位，当*ERCC1*与*XPD*、*XPG*、*XPA*等修复基因将DNA链中受损的部分切除后，DNA链上留下的空缺就由RRM1提供的核苷酸来填补^[12]^。ERCC1被认为与顺铂耐药有关，RRM1与吉西他滨的代谢相关。有研究^[12, 13]^表明，在晚期NSCLC患者中RRM1低表达者的中位OS明显长于高表达者。Simon^[14]^及Olaussen^[15]^报道ERCC1高表达者的术后中位生存期明显长于低表达者。但在晚期NSCLC患者，ERCC1高表达往往导致癌细胞对铂类制剂耐药，使化疗失败。本研究中纳入的52例患者均为RRM1阴性、ERCC1阳性的患者采用GN方案治疗，既避免了含铂方案的耐药，同时RRM1阴性显示吉西他滨的疗效较好。单因素分析发现，检测了RRM1和ERCC1的患者，其PFS较未行检测的患者有延长趋势（*P*=0.058），对影响OS的多因素分析发现，检测RRM1和ERCC1未能明显影响患者的总体生存，这可能和部分患者后续治疗对生存期有影响相关。

本研究是基于国内多中心的回顾性分析，患者时间跨度较大，具有完整临床和生存随访资料的病例数相对较少。同时，由于不同中心采用的RRM1和ERCC1检测手段和技术可能存在差异，因此，对本研究的结果可能产生一定的影响，未来可以进行相关前瞻性研究进一步明确RRM1和ERCC1预测疗效方面的作用。

本研究基于国内多中心的回顾性研究显示，在晚期非小细胞肺癌一线治疗中采用GN方案疗效和安全性均较好，特别是基于ERCC1和RRM1检测基础上，可以做为一种较好的治疗选择。

## References

[1] Jemal A, Siegel R, Ward E (2009). Cancer statistics, 2009. CA Cancer J Clin.

[2] Non-Small Cell Lung Cancer Collaborative Group (1995). Chemotherapy in non-small cell lung cancer: a *meta*-analysis using updated data on individual patients from 52 randomised clinical trials. BMJ.

[3] Non-Small Cell Lung Cancer Collaborative Group (2008). Chemotherapy in addition to supportive care improves survival in advanced non-small-cell lung cancer: a systematic review and *meta*-analysis of individual patient data from 16 randomized clinical trials. J Clin Oncol.

[4] Petrelli NJ, Winer EP, Brahmer J (2009). Clinical Cancer Advances 2009: major research advances in cancer treatment, prevention, and screening--a report from the American Society of Clinical Oncology. J Clin Oncol.

[5] Schiller JH, Harrington D, Belani CP (2002). Comparison of four chemotherapy regimens for advanced non-small cell lung cancer. N Engl J Med.

[6] Gridelli C, Frontini L, Perrone F (2000). Gemcitabine plus vinorelbine inadvanced non-small cell lung cancer: A phase Ⅱ study of three different doses. Br J Cancer.

[7] Laack E, Dickgreber N, Mūlle T (2004). Randomized phase Ⅲ study of gemcitabine and vinorelbine versus gemcitabine, vinorelbine, and cisplatin in the treatment of advanced non-small-cell lung cancer: from the German and Swiss Lung Cancer Study Group. J Clin Oncol.

[8] Plunkett W, Huang P, Xu YZ (1995). Gemcitabine: metabolism, mechanisms of action, and self-potentiation. Semin Oncol.

[9] Johnson SA, Harper P, Hortobagyi GN (1996). Vinorelbine: an overview. Cancer Treat Rev.

[10] Kuzur ME, Shipley DL, Spigel DR (2005). Paclitaxel, carboplatin and gemcitabine (PCG) versus gemcitabine and vinorelbine (GV) in chemotherapy naive patients with advanced non small cell lung cancer: a phaseⅢ trial of the Minnie Pearl cancer research network. J Clin Oncol.

[11] Olaussen KA, Dunant A, Fouret P (2006). DNA repair by ERCC1 in non-small-cell lung cancer and cisplatin-based adjuvant chemotherapy. N Engl J Med.

[12] Ceppi P, Volante M, Novello S (2006). *ERCC1* and *RRM1* gene expressions but not EGFR are predictive of shorter survival in advanced non-small cell lung cancer treated with cisplatin and gemcitabine. Ann Oncol.

[13] Rosell R, Danenberg KD, Alberoija V (2004). Ribonucleotide reductase messenger RNA expression and survival in gemcitabine/cisplatin-treated advanced non-small cell lung cancer patients. Clin Cancer Res.

[14] Simon GR, Sharma S, Cantor A (2005). ERCC1 expression is a predictor of survival in resected patients with non-small cell lung cancer. Chest.

[15] Olaussen KA, Dunant A, Fouret P (2006). DNA repair by ERCC1 in non-small-cell lung cancer and cisplatin-based adjuvant chemotherapy. N Engl J Med.

